# Membrane tension controls the assembly of curvature-generating proteins

**DOI:** 10.1038/ncomms8219

**Published:** 2015-05-26

**Authors:** Mijo Simunovic, Gregory A. Voth

**Affiliations:** 1 Department of Chemistry, Institute for Biophysical Dynamics, James Franck Institute and Computation Institute, The University of Chicago, 5735 S Ellis Avenue, Chicago, Illinois 60637, USA

## Abstract

Proteins containing a Bin/Amphiphysin/Rvs (BAR) domain regulate membrane curvature in the cell. Recent simulations have revealed that BAR proteins assemble into linear aggregates, strongly affecting membrane curvature and its in-plane stress profile. Here, we explore the opposite question: do mechanical properties of the membrane impact protein association? By using coarse-grained molecular dynamics simulations, we show that increased surface tension significantly impacts the dynamics of protein assembly. While tensionless membranes promote a rapid formation of long-living linear aggregates of N-BAR proteins, increase in tension alters the geometry of protein association. At high tension, protein interactions are strongly inhibited. Increasing surface density of proteins leads to a wider range of protein association geometries, promoting the formation of meshes, which can be broken apart with membrane tension. Our work indicates that surface tension may play a key role in recruiting proteins to membrane-remodelling sites in the cell.

## Abstract (web summary)

BAR domain proteins are known to reshape cell membranes. Using coarse-grained molecular dynamics simulations, Simunovic and Voth demonstrate that membrane tension strongly affects the association of BAR proteins, in turn controlling their recruitment to membrane-remodelling sites.

## Graphical abstract

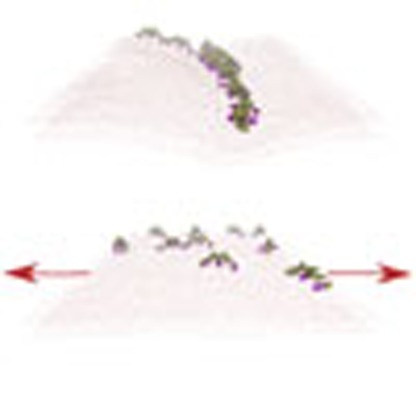

Lipid bilayer membranes are quasi-two-dimensional fluid assemblies that take part in numerous dynamic cellular processes. Their shape is determined by the interplay of molecular interactions at the nanometre-scale lipid–water interface and the macroscopic elastic properties, displayed at its thousand-fold larger area^1^,^2^. In cells, a large number of proteins associate with membranes to alter their shape^3^. This process is a key step in facilitating important tasks such as endocytosis, vesicular trafficking, infection, immune response and the formation of organelles^3^,^4^.

A family of proteins that contain a Bin/Amphiphysin/Rvs (BAR) domain are perhaps the best-known membrane remodellers in cells^5^,^6^. These proteins preferentially bind to curved surfaces and, at sufficiently high membrane-bound densities, they actively remodel the synthetic liposomes and various cellular compartments^7^,^8^,^9^. A large subset of these proteins is termed N-BAR proteins, because they contain an N-terminal amphipathic helix. Electron microscopy imaging demonstrated that N-BAR proteins may polymerize into a cylindrical scaffold that stabilizes the structure of tubules and fixes its radius^10^. It has also been shown that N-BAR proteins can induce fission of the membrane, leading to complex reticular membrane structures^11^ or the disintegration of small liposomes^12^.

The curved shape of BAR proteins provides an intuitive understanding of why they interact with membrane curvature. On the other hand, epsin N-terminal homology domains are not intrinsically curved, but they also sense and induce curvature^13^,^14^,^15^. Epsin N-terminal homology domains interact with the membrane by inserting their amphipathic helices into the bilayer, a process demonstrated to induce significant spontaneous curvature, provided that the insertion is shallow^16^,^17^,^18^,^19^. It has also been predicted that amphipathic helices sense lipid-packing defects^20^,^21^ or in-plane stresses^22^, both of which increase in curved bilayers.

Many studies have shown how molecular interactions and the association of proteins affect the morphology and mechanics of membranes at larger scales. Our aim here is to study the opposite perspective in this relationship: how does membrane mechanics affect the dynamics of protein association at the molecular level? Surface tension is a key mechanical property in regulating the motility and the reshaping of cell membranes. Effective tension in cells is a consequence of (1) pressure difference across the membrane surface and (2) the adhesion of the membrane to the cytoskeleton^23^,^24^,^25^. As tense membranes resist deformations, it is thus expected that tension will affect membrane remodelling. In fact, it has been shown in cells that under high tension the rate of endocytosis decreases^26^ and it can change the molecular sequence of membrane-remodelling events^27^,^28^,^29^. Clearly, surface tension has an important influence on the biochemical pathways of membrane remodelling and the resulting cellular morphology. Interestingly, an experimental paper coinciding with this contribution explored the role of surface tension on the membrane-remodelling power of endophilin. The study demonstrated that the initiation of membrane tubulation is promoted by increased protein density and is inhibited by membrane tension. This result led the authors to conclude that a sudden reduction in membrane tension, for example, due to fusion of exocytic vesicles, could promote the generation of curvature in endocytosis^30^. Moreover, in light of a recently discovered endophilin-mediated endocytic pathway^31^, which relies on actin polymerization for the uptake of, for example, bacterial toxins^32^, it is conceivable that tension could actively modulate the dynamics of membrane-remodelling pathways mediated by BAR proteins.

If we assume that curvature instabilities generate an effective interaction among proteins, we can expect that these interactions will depend on tension, simply based on the membrane's natural length scale, 
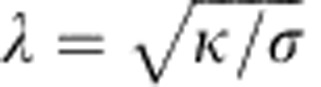
, where *λ* is the curvature decay length, *κ* membrane bending modulus and *σ* membrane tension^33^,^34^,^35^. Indeed, there are numerous analytical studies that have explored how membrane deformations lead to effective interactions among membrane-bound proteins (reviewed in refs 33, 36, 37, 38). The sign, magnitude and length scale of protein–protein interactions depends on their geometry and the way they interact with the membrane. For instance, simultaneous thickening or thinning of the bilayer by two transmembrane inclusions will result in their attraction, whereas if one protein thickens and the other thins the bilayer, they will repel each other^37^. Theory predicts that symmetric inclusions in the membrane will repel each other if they are oriented in the same direction, whereas if they have opposite orientation, they will repel each other at short distances and attract each other at long distances^34^,^35^,^39^,^40^. Furthermore, two particles are predicted to be attractive in case of anisotropic interactions with the membrane^41^, strong membrane affinity^42^,^43^ or high contact angle^44^. It is also suspected that there is a Casimir-like attractive force among particles on fluctuating membranes^45^,^46^,^47^,^48^. Other factors may contribute to protein–protein interactions, such as lipid mixing, lateral protein concentration fluctuations, lipid targeting and so on.

Nevertheless, the way tension influences the collective dynamics of proteins on the membrane has not yet been observed at the molecular level. Moreover, it is not obvious how membrane tension couples with the association of BAR proteins and ultimately how changing membrane tension could quantitatively and qualitatively affect protein-mediated membrane remodelling. In our previous work, we have shown that BAR proteins form linear aggregates and meshes on the membrane^49^, in the same manner—but in a much more robust fashion—as is observed for spherical particles adsorbed on generic fluid membranes^50^. We presume the stronger attractions among proteins are a result of anisotropic local membrane deformations and so we anticipate that membrane tension will play an important role in the large-scale protein assembly. In this work, we use coarse-grained (CG) molecular dynamics (MD) simulations of N-BAR domains as well as spherical particles on planar membranes, at different tensions and protein surface densities. We show that membrane tension has a strong effect on the collective behaviour of proteins, and we provide a visualization of the dynamics of protein assembly as a function of surface tension. Our results imply that surface tension has a more complex effect than just inhibiting large-scale membrane reshaping.

## Results

### Membrane tension inhibits protein–protein interactions

Our recent CG MD simulations showed that N-BAR proteins assemble into string-like aggregates on tensionless membranes^49^. This linear aggregation leads to meshing at a sufficiently high protein surface coverage (∼20%). Here, we carried out CG MD simulations of N-BAR proteins bound to flat membrane sheets at protein coverage ranging from 4 to 20%. Note, the functional N-BAR unit on the membrane is a dimer^51^ and we consider it a single protein molecule, in both the computational modelling and the analysis. Therefore, throughout the text, surface density is defined as percent coverage of N-BAR dimers. Analogously, dimerization and polymerization refer to the association of two or more N-BAR dimers.

By setting a negative external pressure of the simulation barostat, we impose a constant non-zero membrane tension. We note, however, surface tension calculated from the stress tensor in an MD simulation is conjugate to a microscopic area. In tether-pulling experiments, the measured tension is conjugate to the projected membrane^52^. Therefore, the absolute values of surface tension reported here should be taken as approximate and likely systematically higher than the ones obtained in tether-pulling experiments^52^,^53^.

We explored how the change of membrane tension affects the organization of proteins on the surface. We found that when the tension is increased, the dynamics of self-assembly markedly changes. In simulations of tensionless sheets at ∼5% protein coverage, proteins rapidly assemble into linear aggregates, keeping their configuration for the remainder of the trajectory. Increasing the tension to 0.8 mN m^−1^ still favours the assembly along a line, although the configuration of the oligomer is no longer constant and is marked by frequent exchange of dimerization partners. By doubling the tension to 1.6 mN m^−1^, association becomes transient and we no longer observe linear aggregation.

**Figure 1:**
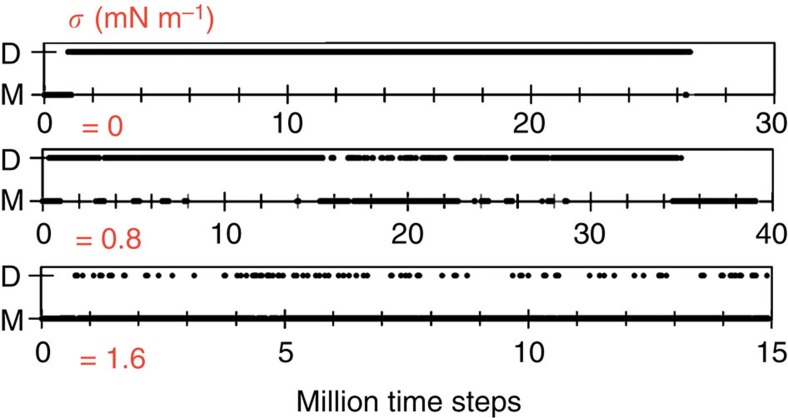
Dynamics of dimerization of N-BARs at 4% surface coverage. The kymogram is generated by observing a protein and recording, at each time step, the dimer (D) or the monomer (M) state. Increasing tension significantly reduces the lifetime of dimers. Each plot is a representative of three individual measurements.

In addition to inhibiting the formation of strings, increased tension reduces the dimerization lifetime. On tensionless membranes, proteins are part of dimers 94% of the trajectory. This lifetime is reduced to 67% at 0.8 mN m^−1^ and further down to 10% at 1.6 mN m^−1^. The kymogram in Fig. 1 depicts how the change in tension alters both the dimerization kinetics and the persistence time of dimers. See also Supplementary Movie 1 showing simulations of N-BAR proteins at zero, intermediate and high membrane tension.

**Figure 2:**
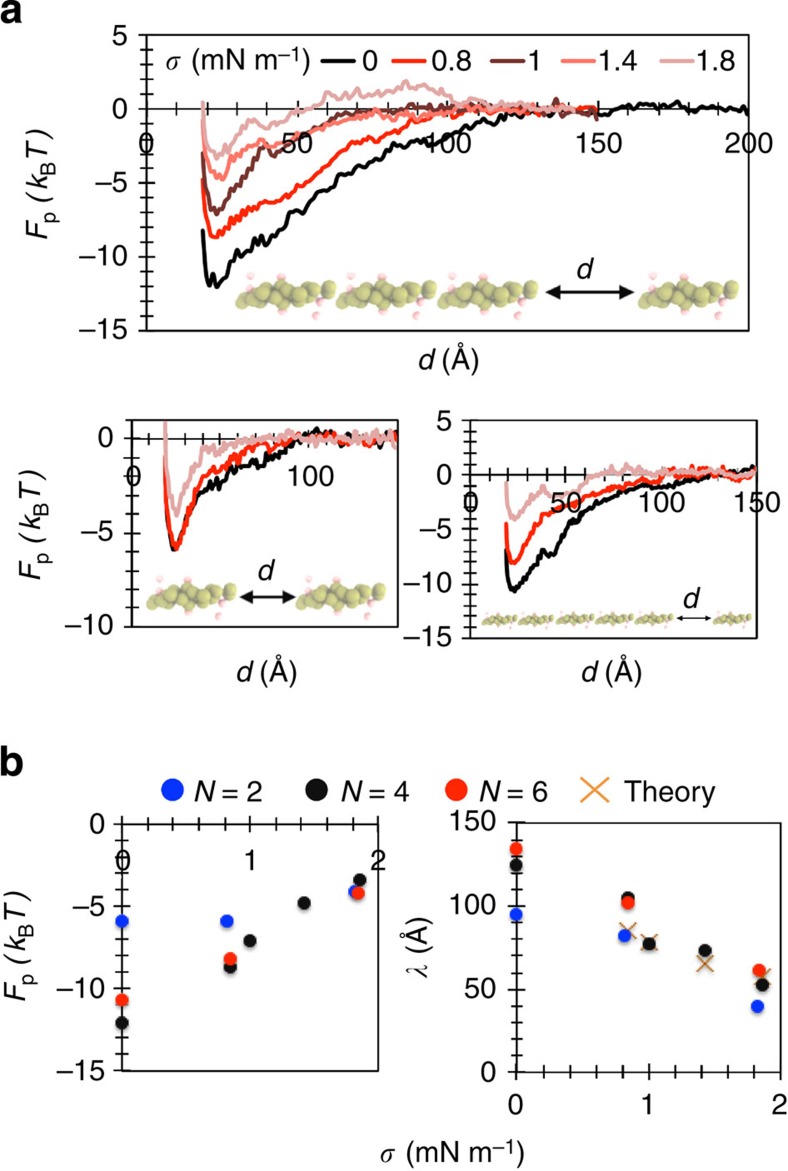
Membrane tension inhibits protein aggregation. (**a**) Polymerization free energy (*F*_p_) as a function of end-to-end distance (*d*) between the incoming N-BAR molecule and a linear chain with: 3 N-BARs, 1 N-BAR or 5 N-BARs, as depicted at the bottom of each plot. For all plots, measurement done from two independent umbrella-sampling calculations, each using 34 different sampling windows (except at zero tension, where 46 windows were used), each window run for 100,000 time steps. (**b**) Magnitude of *F*_p_ and the interaction length scale (*λ*) as a function of membrane tension (*σ*) for the three chain lengths, where *N* is the total number of proteins in the chain. Dots represent free-energy minima taken from measurements in **a**. Cross marks denote values obtained using 
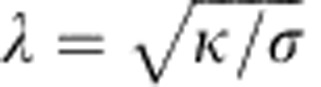
, where *κ*=15 *k*_B_*T*.

To quantify the affinity of proteins to one another, we performed biased MD simulations followed by umbrella-sampling calculations. First, we calculated the energy of an N-BAR molecule joining a preformed aggregate, containing at least three proteins, as a function of the end-to-end distance between the terminal and the joining monomers. We term this free energy the polymerization energy (*F*_p_). We found that with increased tension, the magnitude of *F*_p_ monotonously diminishes from 12 *k*_B_*T* at zero tension to 3 *k*_B_*T* at 1.8 mN m^−1^. If approximating linear dependence of polymerization on membrane tension, the value of *F*_p_ is comparable to the thermal fluctuation energy at >2 mN m^−1^ (Fig. 2a). Considering the absence of imposed significant attractions between N-BAR molecules, the resulting strong effective attraction implies that protein–protein interactions are mediated by local membrane deformations and are, as such, strongly sensitive to membrane tension.

Next, we calculated *F*_p_ as a function of the chain length. The magnitude of *F*_p_ in the course of dimerization has a 6-*k*_B_*T* minimum. If the protein joins a preformed linear aggregate composed of three or five proteins, the magnitude of the energy is increased to, respectively, 12 *k*_B_*T* and 11 *k*_B_*T* (Fig. 2a). This calculation further points to the importance of local membrane curvature in the aggregation of proteins, as local deformations are dependent on the local protein density^54^.

Interestingly, the linear aggregation has a strikingly long interaction range, ∼125 Å for the case of a tetramer (Fig. 2a, top panel), which is 10–20 times the Debye length at physiological ionic strength. This range is comparable to the hypothesized 100-Å average distance between neighbouring proteins on the membrane^33^. Increased membrane tension significantly reduces the interaction range (to ∼50 Å at 1.8 mN m^−1^) (Fig. 2b). Moreover, this range increases with increased chain length, contrary to the magnitude of interaction energy, which seems to converge at *N*>2 (Fig. 2b). As mentioned in the Introduction, the interaction length scale is expected to decrease proportional to the inverse square root of membrane tension, although accurately predicting this length scale from analytical arguments is difficult for a multi-protein linear chain. We compared the maximum interaction length obtained from umbrella sampling to the one calculated based on the natural length scale of the membrane. It appears that there is excellent agreement in the case of only two proteins at all tensions and also in the case of a protein and a linear chain only at high tension, that is, >1 mN m^−1^ (Fig. 2b right, compare dots with cross marks). Evidently, the scaling must diverge at zero tension. However, at low tension, the deviation is likely due to the high anisotropy of the protein, high local curvature induced by a longer linear chain and possibly different scaling of attractions due to membrane fluctuations.

### Tension and protein coverage determine association geometry

**Figure 3:**
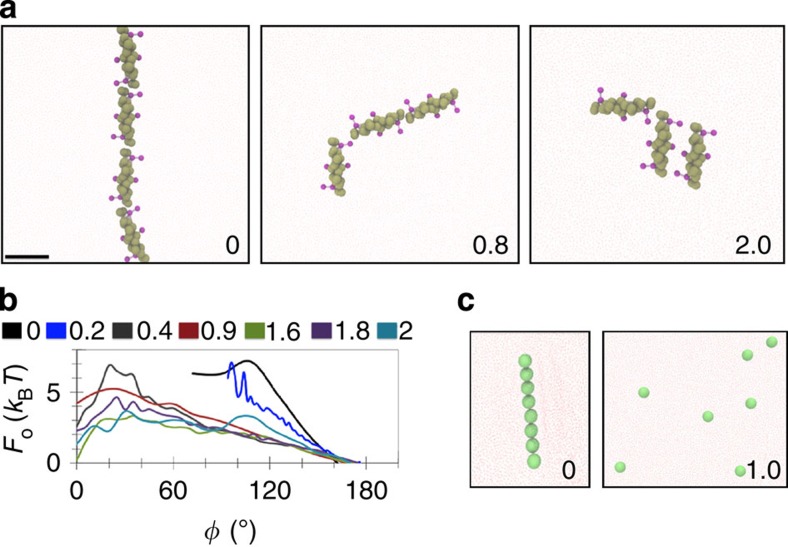
Membrane tension alters N-BAR orientation. (**a**) Self-assembly of N-BAR proteins at different membrane tensions (in mN m^−1^). Scale bar, 10 nm. Each simulation shows a representative snapshot from three simulations, totalling 30 million time steps per configuration. (**b**) Orientation free energy (*F*_o_) as a function of dimer angle (*φ*) at 4% protein coverage, for different membrane tensions (in mN m^−1^). Measured by inverting the Boltzmann distribution of dimerization angles, each plot averaged from simulations in **a**. (**c**) Self-assembly of spherical nanoparticles at different membrane tensions (in mN m^−1^). Panel shows a representative snapshot from two simulations, each run for 10 million time steps.

Increased membrane tension not only weakens protein–protein interactions, but it appears to affect the geometry of their assembly. The N-BAR domain has a characteristic elongated shape, giving rise to two possible in-plane directions of polymerization: end to end (180° between dimers) and side by side (0° between dimers). On tensionless membrane sheets, N-BAR proteins mostly form end-to-end dimers, thus ensuring the longest possible aggregate. To specify, at zero tension, 87% of dimers will align at an angle higher than 160° while only 0.1% will align at an angle lower than 70°. Increasing tension increases the range of the dimerization angles and increasingly favours side-by-side contacts (Fig. 3a).

To quantify the free energy associated with the dimerization angle, we split the range of angles into 5–10° windows, calculated the percent population (in time) of each dimer in the simulation at a given angle window and finally, we computed the free energy by inverting the Boltzmann distribution. We term this value the orientation free energy (*F*_o_). At low tensions (0.2 mN m^−1^ and below), there is a sharp minimum for the formation of end-to-end dimers, with a barrier at ∼7.5 *k*_B_*T* (Fig. 3b). Above 0.2 mN m^−1^, the range of dimerization angles shifts towards smaller values, accompanied by the decrease in the barrier between the two terminal geometries. Finally, at tensions higher than 1.5 mN m^−1^, there is a nearly equal probability of populating both dimerization states (Fig. 3b). It appears that there is a relatively sharp transition between 0.2 and 0.4 mN m^−1^ that allows for the sampling of a wider range of angles of contact, thus allowing for branching of N-BAR aggregates. An increase to 1.6 mN m^−1^ lowers the barrier to ∼4 *k*_B_*T.* Consequently, with increasing membrane tension, there are relatively more side-by-side dimers, although we remind the reader that the overall probability of dimerization is diminished. This change in dimerization geometry indicates that at increased membrane tension, any residual interactions among proteins will tend to maximize their contact surface.

To study the effect of shape of interacting particles on their self-assembly, we tested how spherical particles of size and binding strength comparable to BAR proteins interact with one another at different membrane tensions at ∼10% surface coverage. It has previously been demonstrated that adhesive spherical particles assemble into linear aggregates on a generic fluid membrane model^50^. Provided that particle–membrane affinity is sufficiently high, particles will induce long membrane protrusions^43^. Our CG model reproduces those observations at vanishing tension (Fig. 3c). An increase in tension to 1 mN m^−1^ completely inhibits particle–particle interactions.

**Figure 4:**
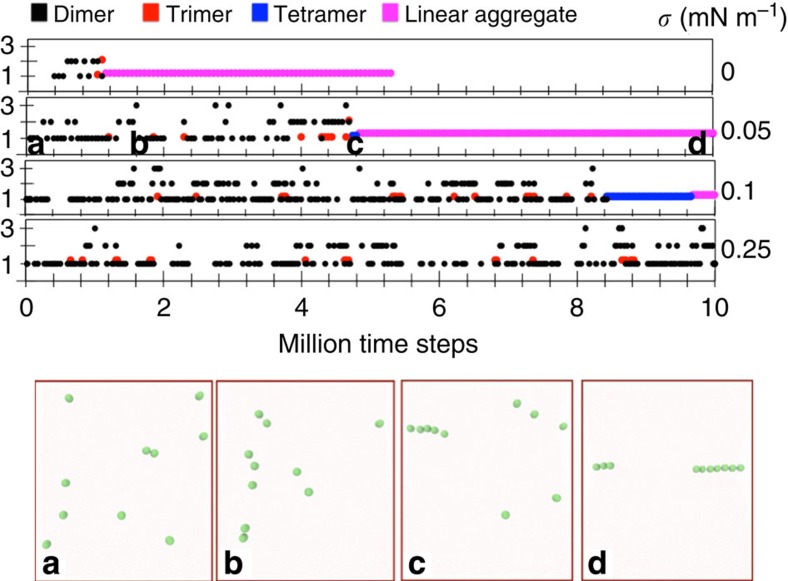
Aggregation dynamics of spherical particles at different tensions. Kymograms show the lifetime of observed dimers, trimers and tetramers of spherical particles, in some cases leading to the formation of a linear aggregate. The *y* axis in all plots represents the number of distinct aggregates. Shown are simulations of particles with 10% membrane coverage, at different membrane tensions, each run for 5 (zero tension) or 10 (non-zero tension) million time steps. Bottom: snapshots from a simulation at 0.05 mN m^−1^ taken at time steps (**a**–**d**) as denoted in the plot above.

By analysing the dynamics of particle association throughout the trajectory at intermediate tensions, we observed that dimers and trimers frequently form, albeit with a short lifetime. Interestingly, a tetramer is the minimum chain that promotes the formation of persistent linear aggregates (Fig. 4). We found that a slight increase in membrane tension, to 0.05 mN m^−1^, appreciably increases the time required for linear aggregation, while at and above 0.25 mN m^−1^, the formation of linear aggregates is completely suppressed (see last kymogram in Fig. 4). Moreover, it is conceivable that pairs of dimers would form in a side-by-side orientation, analogously to N-BAR domains. However, we never observed such association, most likely because pairs of spheres have a too short lifetime to encounter another pair of spheres at the right configuration. These results demonstrate an interesting tension-dependent linear aggregation dynamics of spherical particles but, importantly, they also show that the elongated shape of N-BAR proteins facilitates a wider range of tension-dependent association geometries.

**Figure 5:**
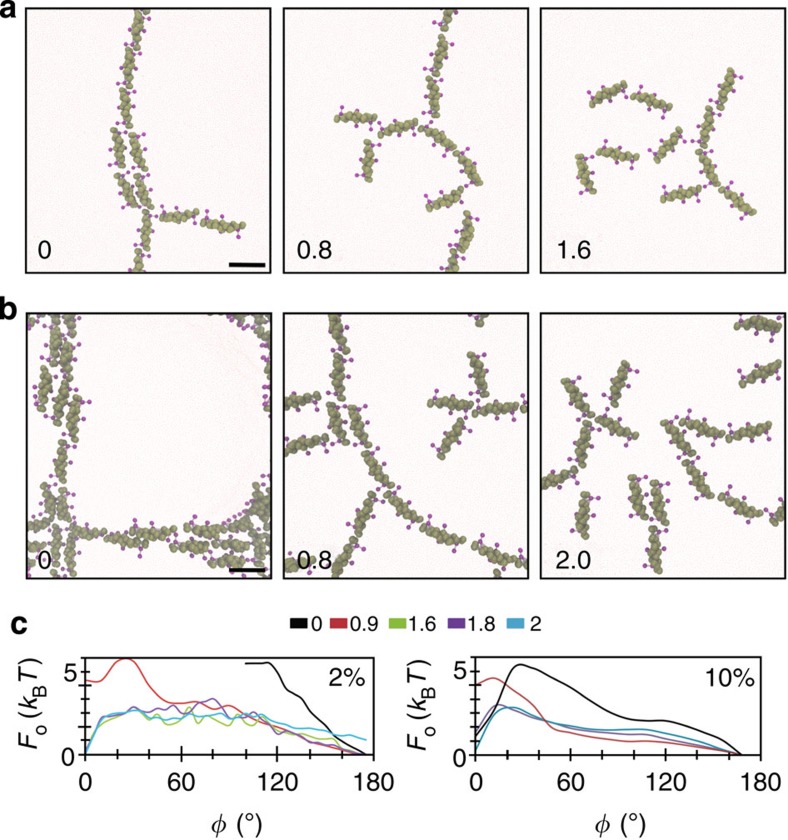
The influence of protein density on self-assembly. Self-assembly at different tensions (in mN m^−1^) at 10% (**a**) and 20% (**b**) N-BAR surface coverage. The scale in all panels is the same. Scale bar, 10 nm (**a**,**b**). Shown are representative snapshots from three simulations, totalling 30 million time steps per configuration. (**c**) The orientation free energy (*F*_o_) at 2% (left) and 10% (right) surface coverage at different membrane tensions (in mN m^−1^). Measured by inverting the Boltzmann distribution of dimerization angles, each plot representing data from three simulations, totalling 30 million time steps per configuration.

An effect on the dimerization geometry similar to increasing tension may be achieved by increasing protein density on the membrane. At 10% surface coverage, we observed branching from the linear aggregate (Fig. 5a), while at 20%, branched strings interconnected to form a meshwork on the surface (Fig. 5b). By calculating the orientation free energy, we found that the barrier between the two dimerization geometries does not in fact decrease in magnitude when increasing the density from 2 to 10%, but it significantly shifts towards smaller angles, thus permitting the sampling of a wider range of angles (Fig. 5c).

Increasing tension to 0.8 mN m^−1^ breaks the meshed structures, although much of the proteins tend to form lines. Finally, high tension (>1.5 mN m^−1^) significantly reduces the protein association, and any residual interactions do not appear to have a preferred angle (Fig. 5).

### Modulating dimerization geometry of BAR domains

To explore how the mechanical parameters of the membrane and the geometric shape of proteins determine their dimerization geometry, we devise very simple scaling arguments. We consider the BAR domain as a spherocylinder bound to an elastic membrane. This model is the simplest approximation of the BAR domain shape and so the arguments derived in this section should be viewed as only qualitative. Nevertheless, despite the many limitations of this model, it seems to provide an intuitive understanding of why some conditions favour side-by-side over end-to-end association (see below). For a more careful treatment, we refer the reader to quantitative analytical models of cylindrical, anisotropic and point-like membrane inclusions, such as in refs 34, 35, 41, 48, 55, 56, 57, 58, 59, 60.

The total free energy of the membrane (*F*) can be written as *F*=*F*_b_+*F*_*w*_+*F*_*σ*_, where *F*_b_ is the membrane bending component, *F*_*w*_ comes from protein–membrane interactions, that is, particle wrapping and *F*_*σ*_ is a consequence of membrane tension, that is, changes in membrane area upon deformation. Local deformation induced by a single protein can be written in terms of the Helfrich bending energy as


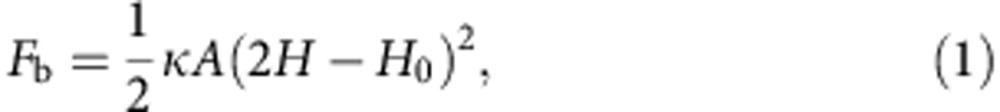


where *κ* is the bending rigidity, *A* the area of the deformation, *H* the mean membrane curvature and *H*_0_ the spontaneous membrane curvature^54^,^61^. *H*_0_ should not appreciably change between two dimerization geometries and we do not take it into account. The bending energy imposed by one spherocylinder of length *L* and radius *R* can be estimated as 
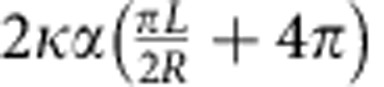
, where *α* is the area fraction of the molecule wrapped by the membrane. We consider that in dimerization, two proteins associate closely enough not to deform the membrane at their interface, based on local curvature calculations carried out previously^49^. Each following term represents the energy per single protein molecule. Bending energies in the end-to-end (*F*_b,e_) and side-by-side (*F*_b,s_) dimerization topologies take the forms:


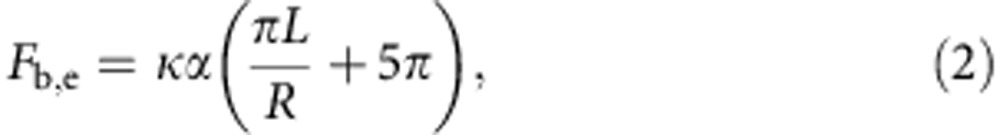



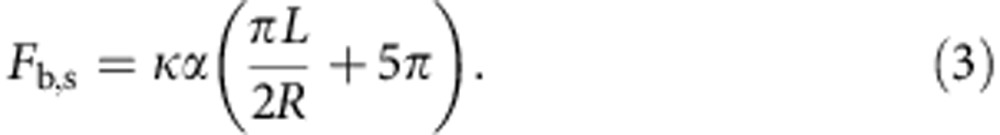


The free-energy gain from particle wrapping can be estimated as adhesion energy 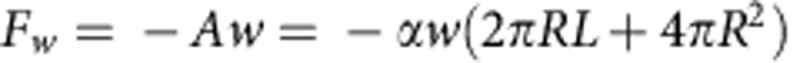, where *w* is the membrane–protein interaction strength per unit area. High values of *w* present a limitation to the model as they would appreciable increase *α*, and so we only consider a narrow range of binding affinities that we demonstrated to lead to linear aggregation of N-BARs^49^. Wrapping energies in the end-to-end (*F*_*w*,e_) and side-by-side (*F*_*w*,s_) dimerization topologies become:


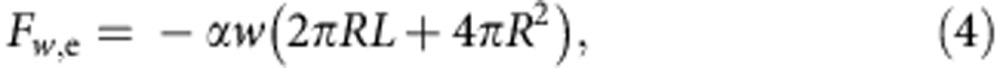



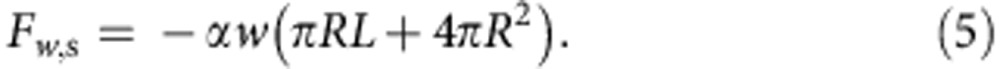


In the case of non-zero surface tension, we add the term associated with the resistance in the change in area, which scales as *σA*. Aggregation of proteins favourably contributes to this energy, as it locally deforms a smaller membrane area per molecule. For the end-to-end and side-by-side geometries, the difference in deformed area compared with a monomer is 4*απR*^2^ and *απRL*, respectively. These are only approximate values and will highly depend on the way proteins corrugate the membrane underneath.

Finally, the net free energy of dimerization in the end-to-end compared with side-by-side configuration is given by


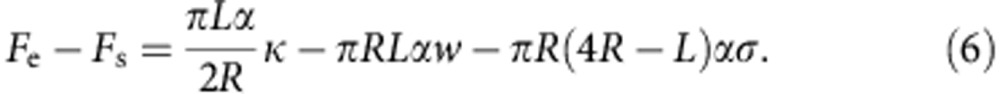


where *F*_e_ and *F*_s_ are total energies of the end-to-end and side-by-side states, respectively. Note, the entropic cost of dimerization cancels out between the two geometries.

**Figure 6:**
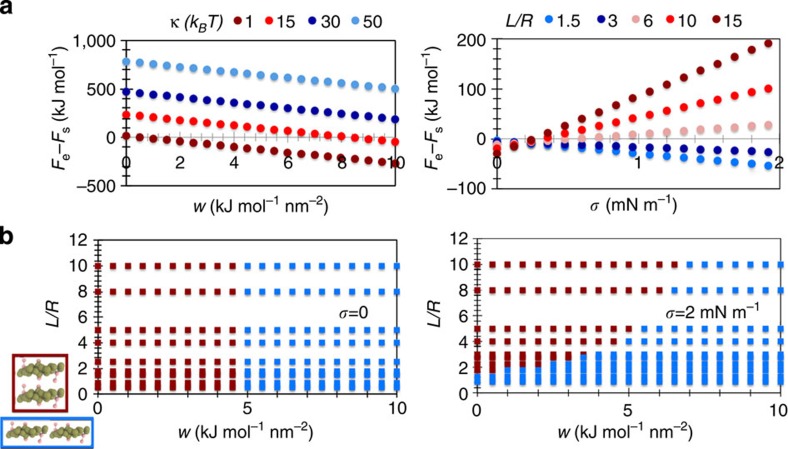
Predicting the dimerization geometry from numerical expressions. (**a**) Free energy of end-to-end (*F*_e_) dimers versus side-by-side (*F*_s_) dimers as a function of membrane–protein interaction strength (*w*) at *σ*=0 (left) and membrane tension at *w*=5 kJ mol^−1^ nm^−2^ (right). End-to-end dimerization is favoured when *F*_e_−*F*_s_<0. (**b**) Configurational diagram relating protein aspect ratio (*L/R*) to *w*, on tensionless and tensed membranes. Red squares: side-by-side dimers, blue squares: end-to-end dimers. In all calculations, we used *α*=0.5, *R*=2.0 nm and *κ*=15 *k*_B_*T* except when these values were varied.

At zero tension, the choice of dimerization topology will be determined by the experimentally tunable parameters, *w* and *κ*. In cells, the affinity of proteins on the membrane is set by the charge density on the BAR domain, the presence of amphipathic helices, the ionic strength of the solvent, membrane composition, lipid sorting and other factors. Bending rigidity is determined by lipid composition. On the basis of our calculations in the previous paragraph, high adhesion strength favours the end-to-end association (that is, *F*_e_*−F*_s_<0), in which the exposed area to the membrane is greater (Fig. 6a). Conversely, bilayers with a higher rigidity will direct the proteins in the side-by-side configuration (Fig. 6a).

Another key parameter that couples with tension in directing the association geometry is the shape of the protein, described by the aspect ratio *L*/*R*. For *L*/*R*>4, higher tension increasingly favours side-by-side geometry (Fig. 6a). Conversely, for *L*/*R*<4, increased tension favours the end-to-end geometry, and *L*/*R*=∼4 is insensitive to membrane tension (Fig. 6a). Interestingly, calculations predict end-to-end geometry at vanishing tension for all *L*/*R*≤3.

Finally, we calculate the configurational diagram that predicts the association geometry from the interaction energy, protein aspect ratio and membrane tension (Fig. 6b). The shape of the configurational diagram changes with increased tension, favouring side-by-side interactions at higher tension, as this configuration is expected to induce less membrane deformation.

The N-BAR domain of endophilin A1, used in this study, has *L*/*R*=6, with *R*=2.0 nm. If we use these parameters in predicting association geometry from phase diagrams in Fig. 6, it appears that our CG MD observations agree very well with the simple scaling arguments presented herein.

## Discussion

Our simulations indicate a significant and geometrically consistent association of N-BAR proteins on the membrane that, in a narrow and biologically realizable space of protein–membrane interaction strengths, leads to linear aggregation^49^. Considering the observed similarity in aggregation between the protein model and simple particles^50^, the linear aggregation phenomenon may apply to a number of proteins that induce curvature instabilities, leading to effective attractions on the membrane. Proteins are more complicated than spherical particles, however, due to their shape and diverse modes of interacting with the membrane. The N-BAR domain is bent and elongated, thus it has multiple ways of forming linear aggregates on the surface. On the basis of previous calculations^49^,^60^,^62^, the attractions among N-BAR are most likely driven by the strong anisotropic interactions with the membrane^41^. We show that, at low membrane tension, N-BAR proteins form aggregates of maximum possible length, thus ensuring rapid and efficient recruitment to membrane-remodelling sites.

When considering a broader range of protein and membrane properties, we predict that side-by-side pairing can be favoured even at low tension, but only on more rigid membranes (for example, those containing cholesterol); as such configuration would bend the membrane less. Conversely, end-to-end pairing is favoured at increased protein–membrane interactions (for example, in case of higher membrane charge or lower ionic strength of the solution) and for more extended proteins (such as F-BAR and I-BAR proteins), which is a consequence of favourable protein–membrane interactions.

Increase in protein density above 10%, as we have shown, permits the branching of the proteins, helping form meshes, that can be broken apart with increased tension. Aside from simple steric arguments, meshing could be a consequence of the inherent repulsion between the lines of protein polymers. Such interactions would likely fix the size of meshes, thereby resulting in a homogenous distribution and radius of tubules, as observed *in vitro*^9^.

At mesoscopic scales, the thermal fluctuations of lipid membranes could act to instantaneously concentrate the proteins at certain domains, while depleting them at others. Areas enriched with N-BAR proteins would serve as nucleation sites for linear aggregation. On the basis of our measurements, an oligomer comprising only three proteins is stable at longer periods of time and generates a strong potential for polymerization. Increasing membrane tension reduces the frequency of membrane fluctuations, and in this way directly inhibits the initiation of protein polymerization.

In cells, membrane tension ranges up to 0.45 mN m^−1^, as measured by a tether-pulling essay in motile keratocytes^25^. This value is already within the range where we see a difference in protein–protein association; in particular, on the association geometry, strength and interaction length scale. Various studies have demonstrated that transient, and in some cases local, increases in membrane tension can significantly impact the sequence of events in membrane remodelling^27^,^28^,^29^. There are several possible sources that could give rise to local membrane tension. One is actin polymerization that increases tension due to adhesive interactions with the membrane. This mechanism is especially important for membrane remodelling that involves endophilin, the N-BAR protein of our study, in light of recent studies demonstrating the interplay of endophilin and actin in a clathrin-independent endocytic pathway^31^,^32^. Moreover, fast scission events (∼1 s)^31^ measured for this endophilin-mediated endocytic mechanism would lead to temporary increases in membrane tension that could affect the association of proteins remaining on the membrane. Conversely, sudden decrease in membrane tension (for example, fusion of exocytic vesicles), would trigger the onset of membrane tubulation, based on recent experiments on endophilin^30^. The same process would promote the formation of linear aggregates from longer distances, again promoting membrane remodelling. In addition, physical limitations (crowding, diffusion barriers) would also impact the in-plane dynamics of lipids. For example, it has recently been demonstrated that several BAR proteins cause lipid diffusion barriers, thereby forming lipid microdomains^63^. Such a barrier would decouple the lipids underneath the proteins from the bulk, effectively giving rise to local membrane tension, which can significantly differ from the low global tension. Therefore, it appears very likely that membrane tension is a crucial modulator of protein self-assembly in cells.

Finally, it would be interesting to investigate how surface tension affects different modes of interaction with the membrane (that is, insertion versus adhesion). In this study, we use highly coarsened models that provide insights into the long-wavelength phenomena. To elucidate the submolecular details of interaction with the membrane and its effect on protein self-assembly, higher-resolution CG models than the one presented here are required and will be part of our future research efforts.

## Methods

### The model

We used CG lipid and protein models and their interaction parameters as described previously^11^,^49^. In brief, three-site lipids were modelled using the hybrid analytical coarse-graining methodology^64^, while the 26-site N-BAR domain was modelled using the elastic-network approach^65^. The protein–membrane attractions were modelled using a Lennard-Jones function set between the protein sites representing the amphipathic helices (six in total) and the lipid head groups, with a 1.8-kcal mol^−1^ well depth, with the minimum set at 1.5 nm. In simulations with nanoparticles, the particles were modelled as incompressible spheres with a 2-nm radius, using Lennard-Jones interactions with lipid head groups, with a 1.0-kcal mol^−1^ well depth, set at 1.5 nm.

### Unbiased simulations

We constructed the systems by randomly arranging N-BAR proteins (or particles) on the surface of a flat membrane patch, 70 nm in *x* and *y* dimensions. We varied the surface density of N-BAR domains from 2 to 20%, while we simulated the nanoparticles at 10% coverage. We carried out the simulations under constant *Np*_*xy*_*T* conditions (the box was allowed to change in size only in *x* and *y* directions, with the *x* and *y* pressure components coupled), using Nosé–Hoover equations of motion, with a coupling constant of 600 *τ* (*τ*∼50 fs). We varied the barostat pressure (*p*_*x*_=*p*_*y*_) between simulations from zero to −4.0 atm, to simulate non-zero membrane tension. We calculated surface tension according to:


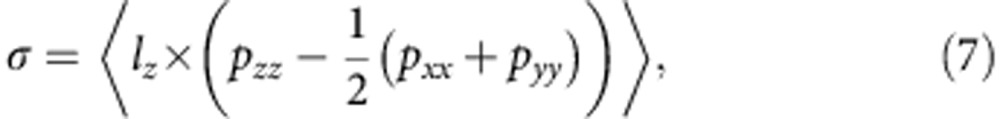


where *p*_*xx*_ and *p*_*yy*_ are tangential components of the pressure tensor, *p*_*zz*_ is the normal component and *l*_*z*_ is the thickness of the bilayer. Note, normally *l*_*z*_ denotes the *z*-dimension of a simulation box, but due to the absence of an explicit solvent, solely the bilayer molecules contribute to pressure. The temperature of the thermostat was set to 300 K, with a coupling constant of 6 *τ*. Systems were initially equilibrated for 1.2 million time steps, then production runs were carried out for 10–30 million time steps (at up to 0.4 *τ*, that is, ∼20 fs per step). All simulations were run using the MD suite LAMMPS^66^.

### Free-energy simulations

To calculate the polymerization free energy, we used the umbrella-sampling approach. We simulated a very large bilayer patch (200 nm in *x* and *y* dimensions) to eliminate the risk of monomers interacting with protein chains in the mirror image. As collective variable, we chose the end-to-end distance between the incoming N-BAR molecule and the terminal N-BAR in the nascent chain. Simulation windows were spaced 1–2 Å and we applied a quadratic potential with a force constant of 1–2 kcal Å^−2^ mol^−1^. Each window was run for 100,000 simulation time steps. We placed additional quadratic constraints to keep the aggregate linear, by keeping the distance between neighbouring N-BARs at 25 Å and an angle of 180°, using a force constant of 0.05 kcal Å^−2^ mol^−1^. Note, in free-energy calculations the effect of these additional constraints cancel out. We repeated each run twice from independent starting trajectories. Finally, we computed the free energy using the weighted histogram analysis method.

## Additional information

**How to cite this article:** Simunovic, M. *et al.* Membrane tension controls the assembly of curvature-generating proteins. *Nat. Commun.* 6:7219 doi: 10.1038/ncomms8219 (2015).

## Supplementary Material

Supplementary Movie 1 Membrane tension strongly inhibits N-BAR assembly. Movie shows CG MD simulations of N-BAR proteins at 10% surface coverage at different membrane tensions, as indicated in each panel. Shown are ~ 20 million time steps.

## References

[b1] Lipowsky R. The conformation of membranes. Nature 349, 475–481 (1991). 1992351 10.1038/349475a0

[b2] Goetz R., Gompper G. & Lipowsky R. Mobility and elasticity of self-assembled membranes. Phys. Rev. Lett. 82, 221–224 (1999).

[b3] McMahon H. T. & Gallop J. L. Membrane curvature and mechanisms of dynamic cell membrane remodelling. Nature 438, 590–596 (2005). 16319878 10.1038/nature04396

[b4] Frost A., Unger V. M. & De Camilli P. The BAR domain superfamily: membrane-molding macromolecules. Cell 137, 191–196 (2009). 19379681 10.1016/j.cell.2009.04.010 PMC4832598

[b5] Qualmann B., Koch D. & Kessels M. M. Let's go bananas: revisiting the endocytic BAR code. EMBO J. 30, 3501–3515 (2011). 21878992 10.1038/emboj.2011.266 PMC3181480

[b6] Mim C. & Unger V. M. Membrane curvature and its generation by BAR proteins. Trends Biochem. Sci. 37, 526–533 (2012). 23058040 10.1016/j.tibs.2012.09.001 PMC3508348

[b7] Peter B. J. *et al.* BAR domains as sensors of membrane curvature: the amphiphysin BAR structure. Science 303, 495–499 (2004). 14645856 10.1126/science.1092586

[b8] Suarez A., Ueno T., Huebner R., McCaffery J. M. & Inoue T. Bin/Amphiphysin/Rvs (BAR) family members bend membranes in cells. Sci. Rep. 4, 4693 (2014). 24796975 10.1038/srep04693 PMC4894391

[b9] Sorre B. *et al.* Nature of curvature coupling of amphiphysin with membranes depends on its bound density. Proc. Natl Acad. Sci. USA 109, 173–178 (2012). 22184226 10.1073/pnas.1103594108 PMC3252917

[b10] Mim C. *et al.* Structural basis of membrane bending by the N-BAR protein endophilin. Cell 149, 137–145 (2012). 22464326 10.1016/j.cell.2012.01.048 PMC3319357

[b11] Simunovic M. *et al.* Protein-mediated transformation of lipid vesicles into tubular networks. Biophys. J. 105, 711–719 (2013). 23931319 10.1016/j.bpj.2013.06.039 PMC3736692

[b12] Boucrot E. *et al.* Membrane fission is promoted by insertion of amphipathic helices and is restricted by crescent BAR domains. Cell 149, 124–136 (2012). 22464325 10.1016/j.cell.2012.01.047 PMC3465558

[b13] Kay B. K., Yamabhai M., Wendland B. & Emr S. D. Identification of a novel domain shared by putative components of the endocytic and cytoskeletal machinery. Protein Sci. 8, 435–438 (1999). 10048338 10.1110/ps.8.2.435 PMC2144257

[b14] Ford M. G. *et al.* Curvature of clathrin-coated pits driven by epsin. Nature 419, 361–366 (2002). 12353027 10.1038/nature01020

[b15] Capraro B. R., Yoon Y., Cho W. & Baumgart T. Curvature sensing by the epsin N-terminal homology domain measured on cylindrical lipid membrane tethers. J. Am. Chem. Soc. 132, 1200–1201 (2010). 20050657 10.1021/ja907936c PMC4205049

[b16] Campelo F., McMahon H. T. & Kozlov M. M. The hydrophobic insertion mechanism of membrane curvature generation by proteins. Biophys. J. 95, 2325–2339 (2008). 18515373 10.1529/biophysj.108.133173 PMC2517036

[b17] Zemel A., Ben-Shaul A. & May S. Modulation of the spontaneous curvature and bending rigidity of lipid membranes by interfacially adsorbed amphipathic peptides. J. Phys. Chem. B 112, 6988–6996 (2008). 18479112 10.1021/jp711107y

[b18] Drin G. & Antonny B. Amphipathic helices and membrane curvature. FEBS Lett. 584, 1840–1847 (2010). 19837069 10.1016/j.febslet.2009.10.022

[b19] Lai C. L. *et al.* Membrane binding and self-association of the epsin N-terminal homology domain. J. Mol. Biol. 423, 800–817 (2012). 22922484 10.1016/j.jmb.2012.08.010 PMC3682188

[b20] Cui H., Lyman E. & Voth G. A. Mechanism of membrane curvature sensing by amphipathic helix containing proteins. Biophys. J. 100, 1271–1279 (2011). 21354400 10.1016/j.bpj.2011.01.036 PMC3043213

[b21] Vanni S. *et al.* Amphipathic lipid packing sensor motifs: probing bilayer defects with hydrophobic residues. Biophys. J. 104, 575–584 (2013). 23442908 10.1016/j.bpj.2012.11.3837 PMC3566459

[b22] Campelo F. & Kozlov M. M. Sensing membrane stresses by protein insertions. PLoS Comput. Biol. 10, e1003556 (2014). 24722359 10.1371/journal.pcbi.1003556 PMC3983069

[b23] Sheetz M. P. & Dai J. Modulation of membrane dynamics and cell motility by membrane tension. Trends Cell Biol. 6, 85–89 (1996). 15157483 10.1016/0962-8924(96)80993-7

[b24] Gauthier N. C., Masters T. A. & Sheetz M. P. Mechanical feedback between membrane tension and dynamics. Trends Cell Biol. 22, 527–535 (2012). 22921414 10.1016/j.tcb.2012.07.005

[b25] Lieber A. D., Yehudai-Resheff S., Barnhart E. L., Theriot J. A. & Keren K. Membrane tension in rapidly moving cells is determined by cytoskeletal forces. Curr. Biol. 23, 1409–1417 (2013). 23831292 10.1016/j.cub.2013.05.063

[b26] Dai J. & Sheetz M. P. Regulation of endocytosis, exocytosis, and shape by membrane tension. Cold Spring Harb. Symp. Quant. Biol. 60, 567–571 (1995). 8824429 10.1101/sqb.1995.060.01.060

[b27] Boulant S., Kural C., Zeeh J. C., Ubelmann F. & Kirchhausen T. Actin dynamics counteract membrane tension during clathrin-mediated endocytosis. Nat. Cell Biol. 13, 1124–1131 (2011). 21841790 10.1038/ncb2307 PMC3167020

[b28] Houk A. R. *et al.* Membrane tension maintains cell polarity by confining signals to the leading edge during neutrophil migration. Cell 148, 175–188 (2012). 22265410 10.1016/j.cell.2011.10.050 PMC3308728

[b29] Masters T. A., Pontes B., Viasnoff V., Li Y. & Gauthier N. C. Plasma membrane tension orchestrates membrane trafficking, cytoskeletal remodeling, and biochemical signaling during phagocytosis. Proc. Natl Acad. Sci. USA 110, 11875–11880 (2013). 23821745 10.1073/pnas.1301766110 PMC3718161

[b30] Shi Z. & Baumgart T. Membrane tension and peripheral protein density mediate membrane shape transitions. Nat. Commun. 6, 5974 (2015). 25569184 10.1038/ncomms6974 PMC4353700

[b31] Boucrot E. *et al.* Endophilin marks and controls a clathrin-independent endocytic pathway. Nature 517, 460–465 (2015). 25517094 10.1038/nature14067

[b32] Renard H. F. *et al.* Endophilin-A2 functions in membrane scission in clathrin-independent endocytosis. Nature 517, 493–496 (2015). 25517096 10.1038/nature14064 PMC4342003

[b33] Phillips R., Ursell T., Wiggins P. & Sens P. Emerging roles for lipids in shaping membrane-protein function. Nature 459, 379–385 (2009). 19458714 10.1038/nature08147 PMC3169427

[b34] Muller M. M., Deserno M. & Guven J. Interface-mediated interactions between particles: a geometrical approach. Phys. Rev. E 72, 061407 (2005). 10.1103/PhysRevE.72.061407 16485947

[b35] Weikl T. R., Kozlov M. M. & Helfrich W. Interaction of conical membrane inclusions: effect of lateral tension. Phys. Rev. E 57, 6988–6995 (1998).

[b36] Goulian M. Inclusions in membranes. Curr. Opin. Colloid Interface Sci. 1, 358–361 (1996).

[b37] Gil T. *et al.* Theoretical analysis of protein organization in lipid membranes. Biochim. Biophys. Acta 1376, 245–266 (1998). 9804966 10.1016/s0304-4157(98)00022-7

[b38] Saric A. & Cacciuto A. Self-assembly of nanoparticles adsorbed on fluid and elastic membranes. Soft Matter 9, 6677–6695 (2013).

[b39] Iglic A. Advances in Planar Lipid Bilayers and Liposomes Elsevier Science (2012).

[b40] Evans A. R., Turner M. S. & Sens P. Interactions between proteins bound to biomembranes. Phys. Rev. E 67, 041907 (2003). 10.1103/PhysRevE.67.041907 12786396

[b41] Dommersnes P. G. & Fournier J. B. N-body study of anisotropic membrane inclusions: membrane mediated interactions and ordered aggregation. Eur. Phys. J. B 12, 9–12 (1999).

[b42] Bahrami A. H., Lipowsky R. & Weikl T. R. Tubulation and aggregation of spherical nanoparticles adsorbed on vesicles. Phys. Rev. Lett. 109, 188102 (2012). 23215335 10.1103/PhysRevLett.109.188102

[b43] Saric A. & Cacciuto A. Mechanism of membrane tube formation induced by adhesive nanocomponents. Phys. Rev. Lett. 109, 188101 (2012). 23215334 10.1103/PhysRevLett.109.188101

[b44] Reynwar B. J. & Deserno M. Membrane-mediated interactions between circular particles in the strongly curved regime. Soft Matter 7, 8567–8575 (2011).

[b45] Bitbol A. F., Dommersnes P. G. & Fournier J. B. Fluctuations of the Casimir-like force between two membrane inclusions. Phys. Rev. E 81, 050903 (2010). 10.1103/PhysRevE.81.050903 20866178

[b46] Goulian M., Bruinsma R. & Pincus P. Long-range forces in heterogeneous fluid membranes. Europhys. Lett. 22, 145–150 (1993).

[b47] Lin H. K., Zandi R., Mohideen U. & Pryadko L. P. Fluctuation-induced forces between inclusions in a fluid membrane under tension. Phys. Rev. Lett. 107, 228104 (2011). 22182045 10.1103/PhysRevLett.107.228104

[b48] Golestanian R., Goulian M. & Kardar M. Fluctuation-induced interactions between rods on a membrane. Phys. Rev. E 54, 6725–6734 (1996). 10.1103/physreve.54.6725 9965898

[b49] Simunovic M., Srivastava A. & Voth G. A. Linear aggregation of proteins on the membrane as a prelude to membrane remodeling. Proc. Natl Acad. Sci. USA 110, 20396–20401 (2013). 24284177 10.1073/pnas.1309819110 PMC3870675

[b50] Saric A. & Cacciuto A. Fluid membranes can drive linear aggregation of adsorbed spherical nanoparticles. Phys. Rev. Lett. 108, 118101 (2012). 22540513 10.1103/PhysRevLett.108.118101

[b51] Capraro B. R. *et al.* Kinetics of endophilin N-BAR domain dimerization and membrane interactions. J. Biol. Chem. 288, 12533–12543 (2013). 23482561 10.1074/jbc.M112.435511 PMC3642301

[b52] Fournier J. B. & Barbetta C. Direct calculation from the stress tensor of the lateral surface tension of fluctuating fluid membranes. Phys. Rev. Lett. 100, 078103 (2008). 18352601 10.1103/PhysRevLett.100.078103

[b53] Fournier J. B., Ajdari A. & Peliti L. Effective-area elasticity and tension of micromanipulated membranes. Phys. Rev. Lett. 86, 4970–4973 (2001). 11384394 10.1103/PhysRevLett.86.4970

[b54] Leibler S. Curvature instability in membranes. J. Phys. 47, 507–516 (1986).

[b55] Mkrtchyan S., Ing C. & Chen J. Z. Y. Adhesion of cylindrical colloids to the surface of a membrane. Phys. Rev. E 81, 011904 (2010). 10.1103/PhysRevE.81.011904 20365396

[b56] Weikl T. R. Indirect interactions of membrane-adsorbed cylinders. Eur. Phys. J. E 12, 265–273 (2003). 15007662 10.1140/epje/i2003-10058-x

[b57] Muller M. M., Deserno M. & Guven J. Balancing torques in membrane-mediated interactions: exact results and numerical illustrations. Phys. Rev. E 76, 011921 (2007). 10.1103/PhysRevE.76.011921 17677508

[b58] Gosselin P., Mohrbach H. & Muller M. M. Interface-mediated interactions: entropic forces of curved membranes. Phys. Rev. E 83, 051921 (2011). 10.1103/PhysRevE.83.051921 21728585

[b59] Weitz S. & Destainville N. Attractive asymmetric inclusions in elastic membranes under tension: cluster phases and membrane invaginations. Soft Matter 9, 7804–7816 (2013).

[b60] Schweitzer Y. & Kozlov M. M. Membrane-mediated interaction between strongly anisotropic protein scaffolds. PLoS Comput. Biol. 11, e1004054 (2015). 25710602 10.1371/journal.pcbi.1004054 PMC4339200

[b61] Helfrich W. Elastic properties of lipid bilayers: theory and possible experiments. Z. Naturforsch. C 28, 693–703 (1973). 10.1515/znc-1973-11-1209 4273690

[b62] Noguchi H. Two- or three-step assembly of banana-shaped proteins coupled with shape transformation of lipid membranes. Europhys. Lett. 108, 48001 (2014).

[b63] Zhao H. *et al.* Membrane-sculpting BAR domains generate stable lipid microdomains. Cell Rep. 4, 1213–1223 (2013). 24055060 10.1016/j.celrep.2013.08.024 PMC4105227

[b64] Srivastava A. & Voth G. A. A hybrid approach for highly coarse-grained lipid bilayer models. J. Chem. Theory Comput. 9, 750–765 (2013). 25100925 10.1021/ct300751h PMC4120858

[b65] Ayton G. S., Lyman E. & Voth G. A. Hierarchical coarse-graining strategy for protein-membrane systems to access mesoscopic scales. Faraday Discuss. 144, 347–357 discussion 445-381 (2010). 20158037 10.1039/b901996k PMC4153359

[b66] Plimpton S. Fast parallel algorithms for short-range molecular-dynamics. J. Comput. Phys. 117, 1–19 (1995).

